# A multi-criteria evaluation of online learning platforms for student engagement and academic performance: an Analytic Hierarchy Process

**DOI:** 10.3389/fpsyg.2026.1906004

**Published:** 2026-10-22

**Authors:** Qinyan Huang, Guanlei Yin

**Affiliations:** 1 School of Business, Guangxi Minzu Normal University, Chongzuo, Guangxi, China; 2 Office of Teaching Quality Monitoring and Development Planning, Guangxi Minzu Normal University, Chongzuo, Guangxi, China

**Keywords:** Analytic Hierarchy Process, content quality, online learning platforms, student engagement, user interface

## Abstract

**Introduction:**

Online learning platforms have become an important component of higher education, yet their effectiveness depends on multiple educational and technological characteristics. This study aimed to determine the relative importance of four criteria for evaluating online learning platforms from graduate students’ perspective.

**Methods:**

We administered a structured questionnaire to 50 graduate students with prior experience in online learning environments. We evaluated Content Quality, User Interface, Accessibility, and Interactivity through pairwise comparisons based on the Analytic Hierarchy Process (AHP). The study aggregated individual judgments, calculated priority weights, and examined the consistency of the pairwise comparisons.

**Results:**

Content Quality received the highest normalized weight (0.416; 41.6%), followed by User Interface (0.297; 29.7%), Interactivity (0.147; 14.7%), and Accessibility (0.140; 14.0%). The results indicate that respondents placed greater relative importance on Content Quality and User Interface when evaluating online learning platforms. The findings show that graduate students assign different levels of importance to educational content, interface usability, interaction, and access when they evaluate online learning platforms. The results provide a quantitative basis for assessing platform characteristics and may help educational institutions and platform developers design and evaluate online learning environments. Further research with larger, more diverse samples is needed to examine whether these priorities apply across institutions and educational contexts.

## 1 Introduction

The advent of online learning platforms has significantly transformed the education landscape, particularly with the onset of the COVID-19 pandemic (Ibrahim et al., 2020; Abdallah and Alriyami, 2022; Ma et al., 2023). According to Lambert and Rennie (2021), the rapid adoption of digital platforms became essential to ensure continuity in education during this global disruption (Lambert and Rennie, 2021). Online learning platforms offer broad access to educational content and learning activities across different locations and devices (Balalle, 2024). Their effectiveness, however, depends not only on access to digital resources but also on the quality of educational content, the usability of the platform interface, accessibility, and the opportunities for student interaction.

Accurately measuring student engagement in online learning environments is crucial for improving academic performance. In traditional learning settings, instructors could visually assess student engagement; however, online platforms rely on data-driven methods to gain similar insights. For instance, Learning Management Systems (LMS) collect “navigation records” to track student activity, giving educators valuable information about how students interact with learning materials. These analytics help identify students who may be at risk of disengagement, a factor associated with lower academic performance and higher dropout rates (Foster and Siddle, 2020). By analyzing student participation and online behavior, educators can design targeted interventions, such as providing timely feedback, adjusting course materials, and promoting student interaction.

The abrupt shift to online learning during the COVID-19 pandemic has underscored substantial challenges in preserving student engagement and academic performance (Lemay et al., 2021). Many students experience difficulties due to reduced face-to-face interaction and personalized feedback, resulting in diminished motivation and suboptimal outcomes (Shroff and Vogel, 2009). Although various digital tools and LMS platforms furnish educators with data on student activities, there is a pressing demand for effective strategies to analyze this data and refine the learning experience. Consequently, a critical question arises: How can online learning platforms be optimized to enhance student engagement and academic achievement in a virtual environment?

Assessing the most critical aspects of online learning platforms that affect student engagement and performance is a complex task (Salas-Pilco et al., 2022). Cheng, Yung-Ming (2020) indicated that the quality of educational content, platform usability, accessibility, and interactivity can shape students’ experiences and learning outcomes in online environments (Cheng, 2020). For example, although Massive Open Online Courses (MOOCs) provide broad access to digital education, maintaining consistent student engagement can remain difficult when interaction and support within the learning environment are limited.

Additionally, a knowledge gap remains in understanding how specific course components, such as multimedia elements, assessments, and peer discussions, influence student performance and retention. A more comprehensive approach is needed to evaluate these factors, combining quantitative analytics and qualitative feedback to enhance the learning experience. Based on the literature, this evaluation selects four criteria: content quality, user interface, accessibility, and interactivity. These criteria represent complementary dimensions of the online learning experience. Content quality concerns the accuracy, relevance, structure, and alignment of educational materials with learning objectives. User interface concerns the platform’s usability and organization for accessing learning resources and completing academic tasks. Accessibility concerns students’ ability to access and use platform resources across different devices, locations, internet conditions, and learner needs. Interactivity concerns opportunities for communication, participation, feedback, and collaborative learning. Together, these criteria provide a focused representation of the principal platform characteristics that directly shape students’ access to, use of, and engagement with online learning resources. The present study does not directly measure academic performance through examination grades, grade point average, course completion, or other objective learning outcomes. Instead, it examines graduate students’ perceptions of the relative importance of platform characteristics that may support student engagement and learning. Therefore, references to academic performance in this study concern the perceived relevance of platform characteristics to learning rather than empirically measured learning gains.

Although several studies have employed the Analytic Hierarchy Process (AHP) to evaluate educational technologies, most have focused on Western or undergraduate contexts and have not systematically validated the normalization and consistency of calculated weights. This study distinguishes itself by applying AHP within the context of Chinese higher education, using data from graduate students across multiple disciplines. It further contributes to the literature by ensuring methodological transparency through verified normalization and consistency testing, thereby producing a robust, context-specific prioritization model for evaluating online learning platforms. This study represents a significant advancement in online education by employing the Analytic Hierarchy Process (AHP) to systematically analyze and rank the critical factors influencing student engagement and academic performance. Unlike previous research, which often lacks a structured methodology for evaluating these factors, this study provides a clear, quantitative assessment of key elements, including content quality, user interface, accessibility, and interactivity. The use of AHP enables detailed, reliable prioritization of these factors, providing educators and policymakers with actionable insights to optimize online learning environments. This research’s novelty lies in its robust methodological approach and its ability to translate complex qualitative judgments into precise, actionable recommendations. This structured evaluation enhances the understanding of what drives student success in online settings and offers practical guidance for improving educational outcomes. By providing a comprehensive analysis and clear ranking of factors, this study offers valuable insights to the academic literature and a practical framework for enhancing online education systems. Building upon the limitations identified in previous AHP and MCDM studies, this research aims to provide a contextually grounded and methodologically transparent evaluation of online learning platforms, thereby addressing both conceptual and empirical gaps in the existing literature.

## 2 Literature review

### 2.1 Online learning platforms

Online learning platforms have become an integral part of the education system in recent years as institutions seek to provide adaptable, scalable, and accessible learning environments. These platforms, including Moodle, Coursera, and Google Classroom, serve as digital spaces that facilitate teaching, learning, and assessment, offering educators and students tools designed to enhance educational experiences and promote student engagement (Sahu, 2024). Moodle, Coursera, and Google Classroom are three popular platforms that cater to different educational needs and contexts. Moodle is an open-source Learning Management System (LMS) known for its flexibility and customization options, allowing educators to create highly tailored learning environments. Coursera is a Massive Open Online Course (MOOC) platform that offers scalable courses from prestigious institutions to support lifelong learning and professional development. Google Classroom, integrated with Google’s suite of productivity tools, is widely used for its simplicity and real-time collaboration features.

*Moodle*, one of the most widely used learning management systems (LMS) globally, is renowned for its flexibility and open-source nature. Its modular design allows for extensive customization, enabling educators to create tailored learning experiences suited to their students’ needs (Mpungose and Khoza, 2022). Moodle supports various activities such as forums, quizzes, assignments, and peer assessments, all aimed at fostering interaction and engagement within a virtual learning environment. Its key strength lies in its ability to integrate numerous third-party plugins and tools, enhancing its functionality. Moreover, Moodle’s analytics tools enable tracking of student participation and progress, providing data to improve course delivery and student outcomes. The platform also supports blended learning, making it suitable for both entirely online and hybrid learning contexts.

On the other hand, *Coursera* operates as a Massive Open Online Course (MOOC) platform, offering courses from top universities and organizations worldwide (Dobrovolska et al., 2019). Coursera provides a wide range of learning resources, including video lectures, interactive quizzes, peer-graded assignments, and discussion forums (Wei and Taecharungroj, 2022). One of Coursera’s key pedagogical advantages is its scalability; it can serve thousands of learners simultaneously, making it an invaluable resource for lifelong learning and professional development. Coursera also utilizes data analytics to personalize learning pathways, offering recommendations based on user progress and preferences. Additionally, Coursera’s partnership model grants learners access to high-quality educational content from prestigious institutions, further increasing its appeal.

*Google Classroom*, popular in K-12 and higher education, integrates with Google’s suite of productivity tools, including Google Docs, Sheets, and Slides (Elder, 2020). It offers an easy-to-use interface that simplifies the management of assignments and communication between educators and students (Kumar et al., 2020). Google Classroom’s real-time collaboration tools are a major strength, enabling students to work on projects and share feedback instantly. The platform also integrates seamlessly with other Google services, providing a comprehensive ecosystem for managing coursework. Google Classroom’s simplicity and accessibility make it particularly appealing for institutions with limited technical resources, as it requires minimal setup and maintenance. As shown in Figure 1, online learning platforms fall into three main types: Learning Management Systems (e.g., Moodle), Massive Open Online Course platforms (e.g., Coursera), and Collaborative Platforms (e.g., Google Classroom). Each category supports different pedagogical objectives, ranging from customizable institutional learning environments to globally scalable open courses and real-time collaborative learning spaces.

**Figure 1:**
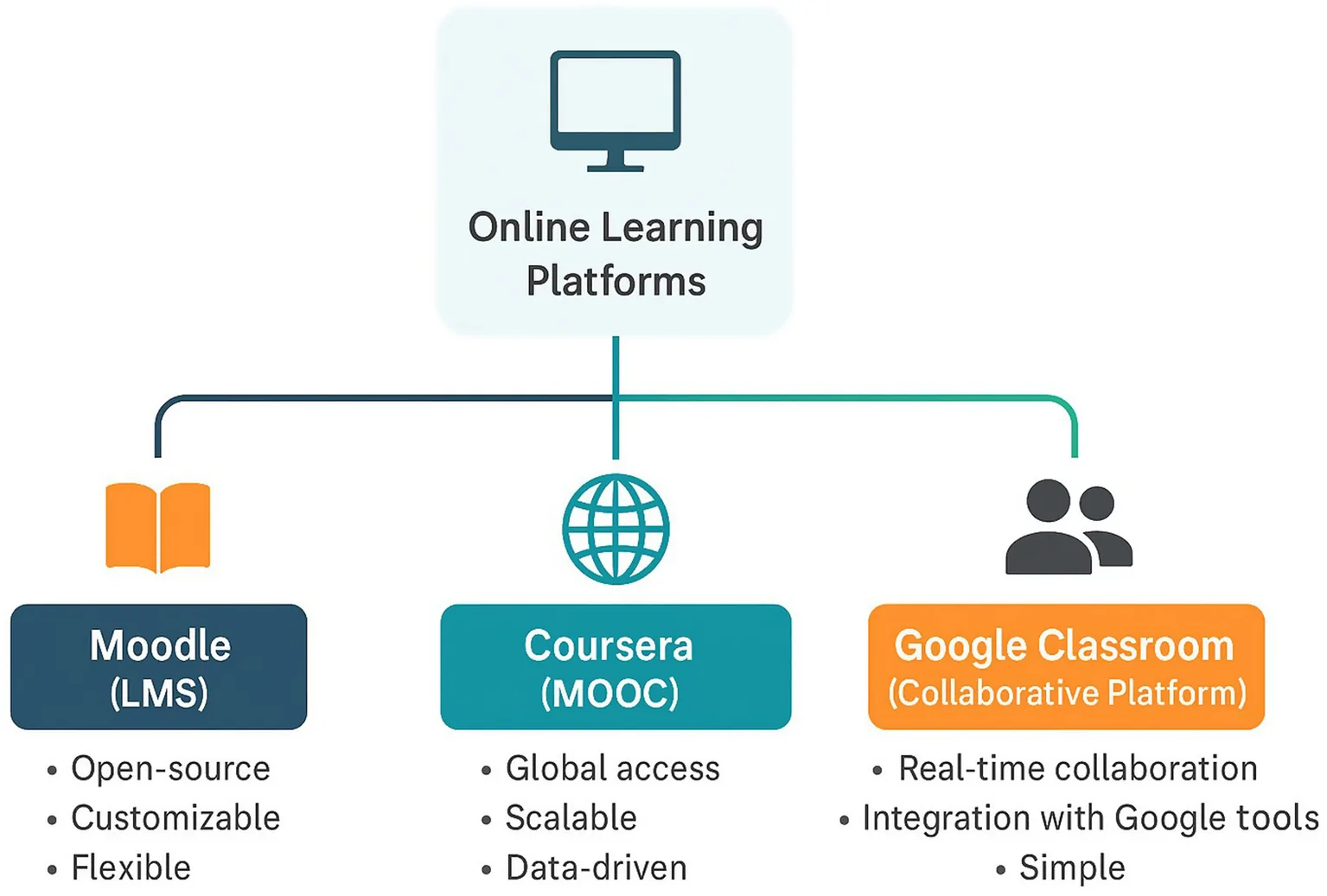
Classification of online learning platforms and their core features.

From a pedagogical perspective, these platforms offer several advantages. They facilitate active learning by providing students with various ways to engage with content, including interactive quizzes, discussion boards, and multimedia resources (Martin and Bolliger, 2018). They also support self-paced learning, which is especially beneficial in asynchronous learning environments where students may require flexibility to study at their own pace (Hew and Cheung, 2014). Furthermore, integrating data analytics into platforms like Moodle and Coursera enables personalized learning experiences, allowing educators to tailor instruction to individual student needs. Table 1 shows the advantages and disadvantages of online learning platforms.

**Table 1:** Advantages and disadvantages of online learning platforms.

Type of Platform	Advantages	Disadvantages
Moodle	- Open-source and customizable - Supports blended and fully online learning - Wide range of plugins and third-party integrations - Third-party integrations - Analytics for tracking student performance and engagement	- Requires technical expertise for customization - Some plugins may introduce compatibility issues
Coursera	- Large-scale, globally accessible platform - Offers courses from top universities - Self-paced learning model, ideal for professional development - Data analytics for personalized learning pathways	- Limited interaction between instructors and students - Certification fees for some courses
Google classroom	- Easy to use and integrates with Google’s productivity tools - Real-time collaboration features - Minimal setup and maintenance required - Ideal for K-12 education	- Limited functionality compared to more advanced LMS platforms - Dependence on Google’s ecosystem

### 2.2 Student engagement in online learning

Student engagement, a multifaceted construct crucial to the effectiveness of online learning environments, encompasses three key dimensions: cognitive, emotional, and behavioral engagement. Understanding these dimensions is crucial in designing effective online learning experiences. Cognitive Engagement relates to students’ mental effort and investment in learning tasks. It involves students’ willingness to think deeply, solve problems, and apply knowledge to new situations (Chen et al., 2019). This type of engagement is associated with higher-order learning processes, such as critical thinking and reflection (Chen et al., 2019). Emotional Engagement encompasses students’ affective responses toward their learning experiences, including feelings of interest, enjoyment, and motivation (Ainley and Ainley, 2011). Emotional engagement significantly impacts students’ attitudes toward learning and their overall motivation (Amiryousefi et al., 2019). Positive emotional engagement can boost students’ persistence and enthusiasm, whereas negative emotions, such as boredom or frustration, can lead to disengagement. Behavioral Engagement involves observable actions indicating students’ participation in learning activities, such as attending classes, completing assignments, and contributing to discussions (Nguyen et al., 2018). This dimension is often measured through attendance records, submission rates, and participation in online forums. Behavioral engagement serves as a proxy for the other two types of engagement and is vital to ensuring students’ active involvement in the learning process. The effectiveness of online learning platforms in promoting student engagement depends largely on their ability to deliver interactive, multimedia-rich experiences and real-time feedback.

Interactivity plays a crucial role in sustaining student engagement. Interactive elements such as quizzes, polls, and discussion boards motivate active participation and make learning more dynamic (Ding et al., 2017). Educators can encourage deeper cognitive engagement and cultivate a more immersive learning experience by involving students in interactive tasks. Multimedia is another critical component that can improve student engagement. Integrating videos, animations, and interactive simulations caters to diverse learning styles and simplifies complex concepts (Lu and Hanim, 2024). Multimedia elements not only capture students’ attention but also bolster cognitive processing by presenting information in multiple formats, promoting better understanding and retention of material (Dunlosky et al., 2013). Real-time feedback furnishes immediate responses to students’ actions and performance, which is pivotal in maintaining engagement. Feedback enables students to recognize their strengths and areas for improvement, fostering a sense of accomplishment and motivation (McMillan and Hearn, 2008). Real-time feedback enables prompt adjustments to learning strategies, keeping students on track and enhancing their overall learning experience.

### 2.3 Criteria for evaluating online learning platforms

#### 2.3.1 Content quality

Content quality refers to the accuracy, relevance, structure, currency, completeness, and alignment of educational materials with intended learning objectives. In online learning environments, students rely heavily on digital materials because direct classroom interaction is reduced. Therefore, the quality of these materials can affect comprehension, cognitive engagement, motivation, and academic achievement. Well-structured content helps students identify learning objectives, follow a topic’s logical development, and connect new information with prior knowledge. Accurate and current materials can also reduce confusion and support reliable knowledge acquisition.

Content quality also concerns the suitability of the format used to present information. Text, video, images, simulations, examples, and assessment materials can represent the same learning objective in different ways. Appropriate multimedia use can support comprehension when complex concepts are presented through suitable visual or interactive resources. In addition, the relevance of learning materials to course objectives matters because students need content that directly supports the knowledge and skills expected in the course. Previous research has therefore associated educational content quality with student satisfaction, engagement, and learning outcomes.

In the present study, content quality is therefore considered a primary criterion because it represents the educational substance delivered through the online platform. Its assessment concerns whether learning resources are accurate, relevant, well-structured, current, and aligned with educational objectives.

#### 2.3.2 User Interface

User interface refers to the visual and functional design through which students interact with an online learning platform (Putri, 2025). It includes navigation structure, page organization, menus, dashboards, resource access, assignment submission, and the presentation of learning information. A clear interface can reduce the effort required to locate resources and complete academic tasks. When navigation is simple and consistent, students can focus more on course activities rather than on understanding how the platform works.

Interface quality also affects the efficiency of learning activities (Putri, 2025). Clear layouts, logical navigation paths, readable information, and accessible functions can support students when they move between lectures, assignments, assessments, and communication tools. In contrast, complex navigation and poorly organized information can create unnecessary cognitive demands and may discourage platform use. Previous research has associated interface usability with student satisfaction, engagement, and learning efficiency.

In this study, user interface is therefore selected as a criterion because it represents the usability dimension of online learning platforms. The criterion concerns the platform’s clarity, organization, navigation, and ease of use from the student perspective.

#### 2.3.3 Accessibility

Accessibility concerns the extent to which students can access and use online learning resources regardless of device, location, internet conditions, or individual learning needs (Papadopoulos et al., 2025). Accessibility concerns the extent to which students can access and use online learning resources regardless of device, location, internet conditions, or individual learning needs. Online learning platforms should provide access through commonly used devices such as desktop computers, tablets, and smartphones. Reliable access is particularly important when students have different levels of technological resources or internet connectivity.

Accessibility also includes design features that support students with disabilities. Examples include compatibility with screen readers, alternative text for visual materials, adjustable text size, keyboard navigation, and well-presented multimedia resources. Such features can reduce barriers to participation and support equitable access to educational activities. Accessibility therefore extends beyond technical availability. It also concerns whether students can use the available resources effectively.

Hamzah et al. (Hamzah et al., 2025) have shown that accessibility can influence participation and the quality of students’ online learning experiences. Access difficulties can interrupt learning activities, reduce participation, and limit students’ ability to use course resources (Ayanwale et al., 2025). In the present study, accessibility is therefore selected as a criterion because continuous, equitable access is a necessary condition for effective participation in online learning.

#### 2.3.4 Interactivity

Interactivity refers to the opportunities an online learning platform provides for students to participate, communicate, receive responses, and collaborate during learning activities (Akram and Abdelrady, 2025; Romero-Saritama et al., 2025). Discussion forums, quizzes, polls, peer assessment, and collaborative activities can support behavioral and cognitive engagement. As discussed in Section 2.2, interactive functions can increase participation and give students opportunities to assess their understanding. In the present study, interactivity is therefore retained as a criterion because it represents the participatory dimension of the online learning environment.

### 2.4 Academic performance in online learning

Academic performance is an important outcome for evaluating online learning environments. Although learner characteristics, instructional practices, and learning analytics can influence academic achievement, this study focuses on platform characteristics that directly shape students’ online learning experiences. Accordingly, the AHP model evaluates content quality, user interface, accessibility, and interactivity as the four criteria.

*Self-regulation* refers to students’ ability to manage their learning processes, encompassing goal setting, time management, and self-monitoring. It is crucial for success in online learning environments, where students often have greater autonomy and less direct supervision than in traditional classroom settings (Eggers et al., 2021). Self-regulated learners actively set specific learning goals, plan and organize their study time, and employ effective strategies to monitor their progress (Zimmerman et al., 2023). Efficient self-regulation also involves staying motivated and overcoming distractions, both of which are vital for maintaining engagement and achieving academic goals online. *Learning analytics* entails collecting, measuring, analyzing, and reporting data about learners and their contexts to enhance learning and educational outcomes (Viberg et al., 2018). Learning analytics provides valuable insights into student behaviors, performance patterns, and engagement levels in online learning environments. By examining data on login frequency, activity participation, and assessment results, educators can identify struggling students and intervene early to provide additional support. Learning analytics also helps personalize learning experiences by adapting content and resources to individual needs and performance, thereby boosting the effectiveness of online instruction (Bienkowski et al., 2012). *Feedback mechanisms* are fundamental to the learning process, providing students with information regarding their performance and areas for improvement (Carless and Boud, 2018). In online learning environments, feedback can be delivered through automated quizzes, instructor comments, peer reviews, and self-assessment tools. Effective feedback is timely, specific, and actionable, helping students understand their strengths and weaknesses and adjust their learning strategies. Real-time feedback can significantly affect academic performance by helping students address misunderstandings and quickly refine their knowledge and skills.

### 2.5 Analytic Hierarchy Process (AHP) in educational research

The Analytic Hierarchy Process (AHP) is a structured decision-making methodology that solves complex problems by breaking them into simpler components. Developed by Saaty (1980), the AHP facilitates decision-making through a multi-level hierarchical structure that represents a problem’s various elements. Table 2 illustrates AHP applications in the educational context.

**Table 2:** Applications of AHP in educational concepts.

Authors	Ref	Year	Scope	Aim	Results	Conclusions
Chen et al.	Chen et al. (2013)	2013	Energy education framework incorporating carbon reduction	Enhance energy literacy by developing a framework and prioritizing indicators	- Key goals: “civic responsibility for a sustainable society” and “low-carbon lifestyle.” - Top indicators: “awareness and self-efficacy.”	The framework effectively highlights key goals and indicators for enhancing energy education.
Farid et al.	Farid et al. (2015)	2015	E-learning challenges in HEIs in developing countries	Create a hierarchical model of challenges and recommend strategies for e-learning integration	- Identified 17 critical challenges across 5 dimensions	Provides a roadmap for overcoming e-learning barriers in developing regions.
Ahmad and Hussain	Ahmad and Hussain (2017)	2017	Factors motivating African students in UAE higher education	Identify key influences using push-pull factor theory and AHP	- Crucial factors: learning environment and geographic proximity - Other elements: moderate impact	Enhances understanding of factors influencing African students and offers insights for recruitment.
Thanassoulis et al.	Thanassoulis et al. (2017)	2017	Evaluating higher education teaching performance	Enhance teaching quality using AHP and DEA	- Comprehensive assessment of teaching performance - Identified areas for instructional improvement	The method provides a thorough evaluation of teaching performance and areas for improvement.
Ponsiglione et al.	Ponsiglione et al. (2022)	2022	Quality assessment in medical education using hybrid decision-making	Augment training quality by evaluating user satisfaction and prioritizing program parameters	- Successful in identifying areas requiring improvement - Enhanced overall training quality	The hybrid method improves quality assessment and training enhancement.

Previous studies employing the Analytic Hierarchy Process (AHP) and other Multi-Criteria Decision-Making (MCDM) methods in educational contexts have produced varying results regarding the relative importance of evaluation criteria (Chen et al., 2013; Farid et al., 2015; Ahmad and Hussain, 2017; Thanassoulis et al., 2017; Ponsiglione et al., 2022). Several studies have identified content quality as the dominant factor influencing learner satisfaction, while others have emphasized usability, accessibility, or interactivity, depending on the institutional or cultural context. However, many of these works remain methodologically limited, offering minimal discussion of normalization accuracy or validation of the consistency ratio. Moreover, most existing research has focused on undergraduate or Western learning environments, leaving graduate-level and non-Western perspectives underexplored. To address these gaps, the present study employs a rigorously verified AHP approach to assess the relative importance of content quality, user interface, accessibility, and interactivity in evaluating online learning platforms in a Chinese higher education context. This critical synthesis establishes a clear conceptual linkage between prior findings and the current research objectives, reinforcing both the study’s contextual relevance and methodological contribution.

## 3 Methodology

### 3.1 Selection of criteria

The four evaluation criteria used in this study were selected from the literature on online learning effectiveness, student engagement, and academic performance. The literature review indicates that online learning quality depends on both the educational resources provided and the characteristics of the digital environment through which students access and use these resources (Ayanwale et al., 2025; Munna et al., 2024; Santiago et al., 2021). Content quality represents the educational value and relevance of learning materials, while user interface represents platform usability and ease of navigation. Accessibility refers to students’ ability to access and use learning resources under different technical and individual conditions. Interactivity represents opportunities for participation, communication, collaboration, and response within the learning environment. These four criteria are therefore selected because they represent distinct but related dimensions of the online learning platform that can affect students’ engagement and learning experience.

The selection is also guided by the need to construct a focused AHP hierarchy. Other factors reported in the literature, such as flexibility, scalability, feedback mechanisms, self-regulation, and learning analytics, are not treated as separate criteria in the present model. Flexibility and scalability describe broader characteristics or advantages of online education systems, while feedback is treated as a feature that can occur within interactive learning activities. Self-regulation represents a learner characteristic, and learning analytics represents an analytical and instructional support function. These concepts are therefore acknowledged in the literature review but are excluded from the AHP criteria to maintain a clear and non-overlapping evaluation structure. Based on this literature-based selection process, the AHP model evaluates four criteria: Content Quality, User Interface, Accessibility, and Interactivity (Veljić et al., 2025; Gümüshan and Çakir, 2023; Atıcı et al., 2022).

- User Interface (UI)

The user interface of an online learning platform refers to the system’s design and layout through which users—students and educators—interact with educational content and features. A well-designed UI is crucial for enhancing the user experience, as it enables easy navigation and seamless interaction with the platform’s tools and resources. Platforms with intuitive, user-friendly interfaces reduce cognitive load, allowing students to focus on learning rather than struggling with the system’s technicalities. Research has shown that ease of use significantly impacts student engagement, with easy-to-navigate, understandable platforms leading to higher participation and academic success. In addition, a clear, structured interface supports learners in efficiently accessing resources and managing assignments, which contributes to better time management and educational outcomes (Abuhassna et al., 2020).

- Interactivity

Interactivity is one of the most essential elements in fostering student engagement in online learning environments. Interactive features such as discussion forums, quizzes, polls, peer assessment, and collaborative activities encourage active participation and give students opportunities to receive feedback on their learning activities. Platforms like Moodle and Coursera offer interactive tools that promote engagement by enabling learners to collaborate, discuss, and test their understanding through peer assessments and group projects. Moreover, real-time feedback allows students to assess their performance and make necessary adjustments immediately, a factor strongly correlated with improved academic performance. Interactive multimedia resources such as videos, simulations, and animations also enhance engagement by catering to different learning styles and improving retention of complex concepts (Abuhassna et al., 2020; Sharif Nia et al., 2023).

- Content Quality

Content quality is a critical determinant of engagement and academic performance in online learning platforms. High-quality educational content is well-structured, accurate, up to date, and aligned with learning objectives. It should cater to diverse learning preferences by incorporating formats such as text, video, and multimedia. Platforms like Coursera, which partner with leading universities and organizations, offer students high-caliber, globally recognized educational content. The relevance and depth of content directly influence a student’s cognitive engagement by promoting critical thinking, problem-solving, and application of knowledge to real-world scenarios. Furthermore, comprehensive content encourages self-paced learning, a feature that can significantly enhance academic outcomes in asynchronous learning environments (Sharif Nia et al., 2023).

- Accessibility

Accessibility refers to how easily students can access learning materials and platform functionalities, regardless of their geographical location, device type, or internet connection (Munna et al., 2024). It also encompasses accessibility features for students with disabilities, such as screen readers, alternative text, and adjustable font sizes (Santiago et al., 2021). Accessible platforms ensure that all students, including those with limited resources or special needs, can participate fully in the learning process (Nyongesa and Van Der Westhuizein, 2025). A platform’s ability to work efficiently across devices (such as smartphones, tablets, and desktops) and under diverse internet conditions is crucial to ensuring uninterrupted access to educational materials. Studies indicate that accessibility challenges can hinder student engagement and negatively affect academic performance, underscoring their importance as an evaluation criterion for online learning platforms.

Based on the literature reviewed above, four criteria are selected for the AHP evaluation: Content Quality, User Interface, Accessibility, and Interactivity. These criteria represent the educational, usability, access, and participation dimensions of an online learning platform. The AHP procedure evaluates each criterion to determine its relative importance. Figure 2 presents the conceptual structure of the AHP evaluation.

**Figure 2:**
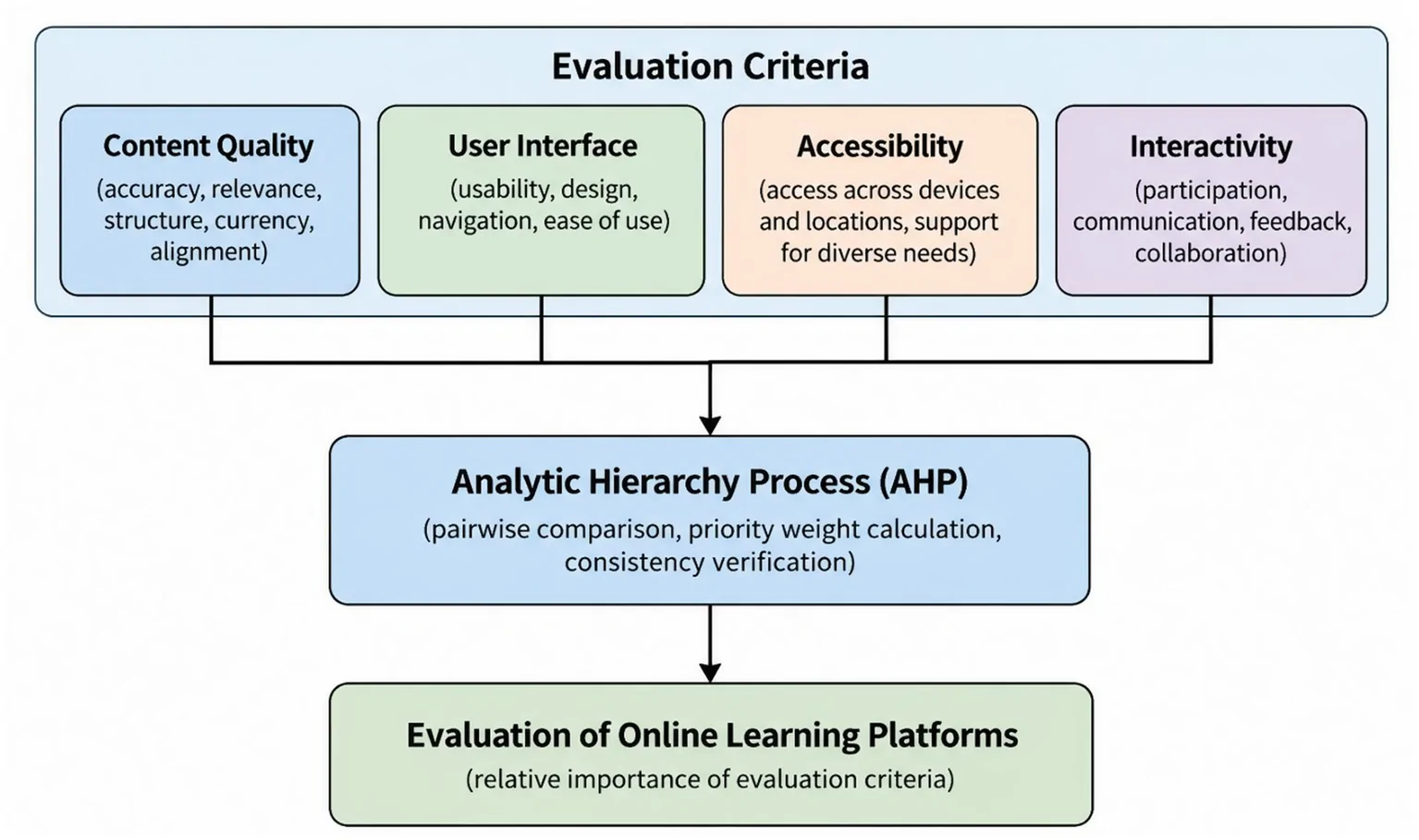
Conceptual framework for evaluating online learning platforms using the Analytic Hierarchy Process (AHP).

### 3.2 Data collection through questionnaires

The primary data for this study were collected using a structured questionnaire administered to 50 graduates from Guangxi Minzu Normal University in China. All procedures followed institutional ethical standards, and researchers obtained written informed consent from all participants before data collection. Participants had experience with online learning platforms and rated the four selected criteria: User Interface, Interactivity, Content Quality, and Accessibility. The sample included graduate students from diverse academic disciplines, including engineering, management, education, and the social sciences, ensuring multiple academic perspectives and minimizing potential disciplinary bias in the responses. We employed a discipline-stratified convenience sampling approach to ensure representation across major academic fields. We defined four strata based on disciplinary groupings within the university: engineering, management, education, and social sciences. We distributed invitations through departmental mailing lists and graduate program coordinators. We invited 68 students, of whom 50 completed the questionnaire, for a response rate of 73.5%. Participants had prior experience using online learning platforms in coursework. Most respondents reported at least 1 year of experience with online learning environments, and the most used platforms included Moodle, Coursera, and Google Classroom. These characteristics provide contextual grounding for interpreting the judgments used in the AHP analysis. The sample included graduate students from different stages of study, ensuring variation in familiarity with online learning environments and instructional platforms.

The data collection process involved distributing the questionnaire digitally, providing convenience and anonymity for the participants. Graduates were selected to reflect a diverse range of disciplines and educational backgrounds, enhancing the robustness of the findings. Once the responses were gathered, the data were systematically processed and formatted for input into the Analytic Hierarchy Process (AHP) model. The numerical responses from the questionnaire enabled the development of a pairwise comparison matrix, which was used to calculate the relative importance of the criteria and form the basis for the hierarchical analysis and subsequent prioritization. Figure 3 illustrates the step-by-step process of designing the questionnaire, collecting data, and integrating it into the Analytic Hierarchy Process (AHP). The process starts with identifying the research objectives, defining the evaluation criteria, designing the questionnaire, collecting responses from 50 graduates, and formatting the data for AHP analysis.

**Figure 3:**
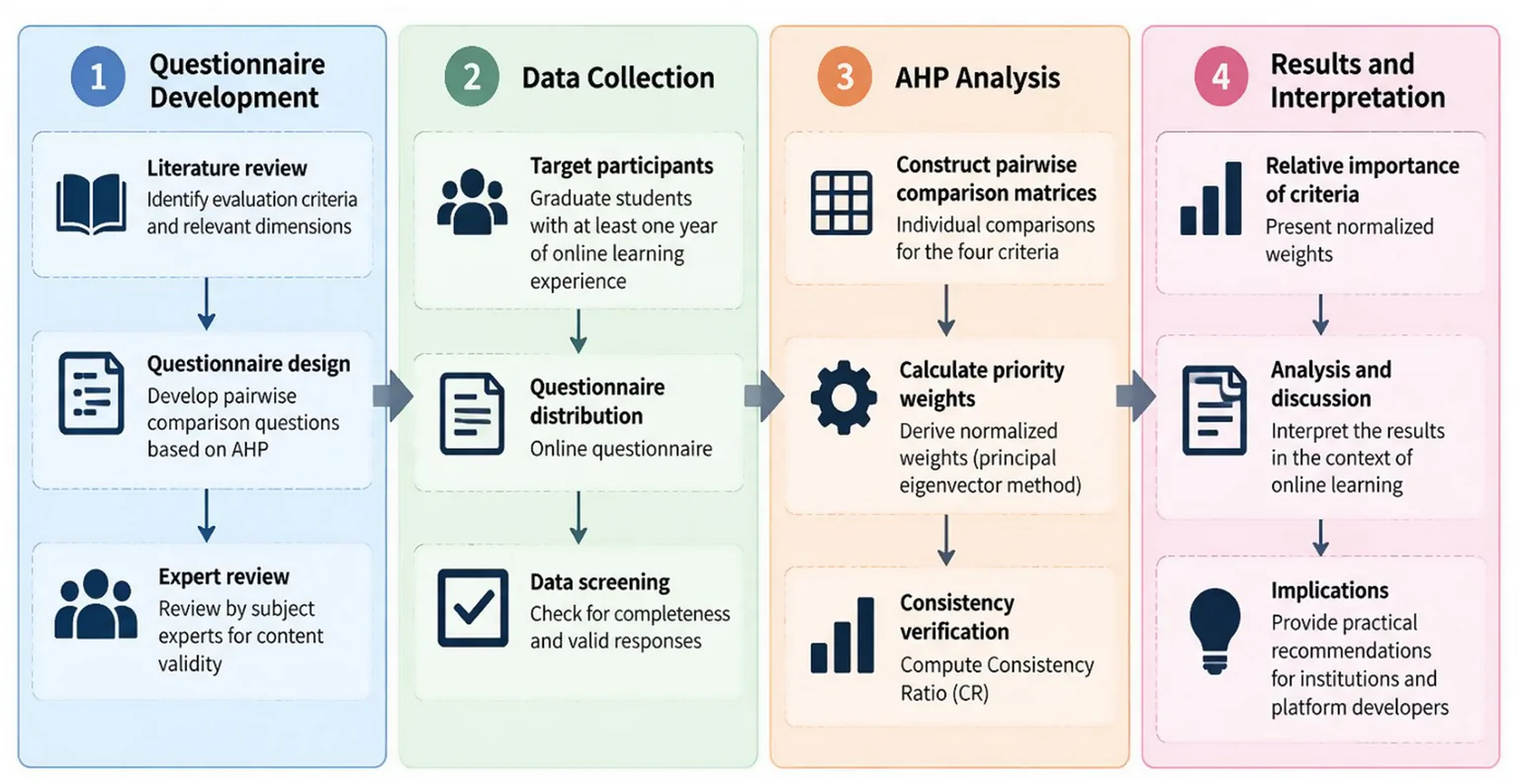
Analytical Hierarchy Process (AHP) workflow illustrating the sequential steps of pairwise comparisons, weight derivation, and consistency verification used to determine the relative importance of evaluation criteria.

Each questionnaire required participants to conduct pairwise comparisons among the four criteria: Content Quality, User Interface, Accessibility, and Interactivity, using the nine-point AHP scale (1 = equal importance, 3 = moderate importance, 5 = strong importance, 7 = extreme importance, and 9 = extreme importance). For each pair of criteria, respondents indicated which factor they considered more important and to what degree. The individual responses were then converted into pairwise comparison matrices, one for each participant. To obtain a single, representative group judgment, the 50 individual matrices were aggregated by computing the geometric mean of corresponding elements, in line with Saaty’s (1980) group AHP approach. This aggregated matrix served as the basis for the pairwise comparison matrix shown in Table 3, from which the normalized priority weights in Tables 4 and 5 were derived.

**Table 3:** Pairwise comparison matrix of evaluation criteria used in the Analytic Hierarchy Process (AHP).

Criteria	User interactivity	Interactivity	Content quality	Accessibility
User interface	1	3	1/2	2
Interactivity	1/3	1	1/2	1
Content quality	2	2	1	3
Accessibility	1/2	1	1/3	1

**Table 4:** Normalized Pairwise Comparison Matrix of Evaluation Criteria in the AHP Model.

Criteria	User interface	Interactivity	Content quality	Accessibility
User interface	0.261	0.429	0.214	0.286
Interactivity	0.087	0.143	0.214	0.143
Content quality	0.522	0.286	0.429	0.429
Accessibility	0.130	0.143	0.143	0.143

**Table 5:** Normalized priority weights of evaluation criteria derived from the AHP Analysis.

Criterion	Weight	Normalized weight	Percentage (%)
Content quality	0.416	0.416	41.6
User interface	0.297	0.297	29.7
Interactivity	0.147	0.147	14.7
Accessibility	0.140	0.140	14.0
Total	1.000	1.000	100.0

Subsequent steps include performing AHP calculations, interpreting the results, and generating actionable recommendations for stakeholders. After collecting the completed questionnaires, we used each respondent’s answers to construct a pairwise comparison matrix reflecting their judgments of the relative importance among the four criteria: Content Quality, User Interface, Accessibility, and Interactivity.

The questionnaire employed Saaty’s (1980) standard 1–9 scale, where 1 indicated equal importance, and 9 indicated extreme importance of one criterion over another. To obtain a single group judgment, the 50 individual matrices were combined using the geometric mean of corresponding entries, which is the recommended approach for aggregating multiple evaluators’ AHP inputs. The aggregated matrix represents the collective preferences of all participants and serves as the foundation for subsequent AHP calculations. The questionnaire was researcher-developed rather than directly adopted from a previously validated instrument. The questionnaire content was derived from the four evaluation criteria identified through the literature review: Content Quality, User Interface, Accessibility, and Interactivity. The pairwise comparison structure followed the standard AHP procedure and Saaty’s nine-point comparison scale. Each respondent compared the relative importance of the four criteria in pairs. We addressed content validity by deriving the four criteria from the literature reviewed in Sections 2.3 and 3.1 and ensuring each criterion represented a distinct dimension of online learning platform evaluation. We then developed the questionnaire structure according to the established AHP pairwise comparison procedure. Because the instrument collects pairwise judgments rather than responses on a psychometric Likert scale, we did not use internal consistency measures such as Cronbach’s alpha. Instead, we assessed judgment consistency using the AHP Consistency Ratio. The resulting CR of 0.038 was below the recommended threshold of 0.10, indicating acceptable consistency in the aggregated judgments.

In group-AHP studies, the emphasis is on expert/judge diversity and judgment consistency rather than probabilistic power; panels of 10–30 are common in the literature. Our *N* = 50 exceeds this range, increasing the stability of the aggregated pairwise comparisons (geometric mean). The resulting matrix produced an acceptable Consistency Ratio (CR = 0.038 < 0.10). Additionally, a ± 1 step sensitivity check on key pairwise entries did not alter the criteria rank order, indicating that the panel size was adequate to derive stable priority weights.

Respondents were required to have prior experience with online learning platforms because the AHP questionnaire required comparative judgments about content quality, user interface, accessibility, and interactivity. Respondents were required to have prior experience with online learning platforms because the AHP questionnaire required comparative judgments about content quality, User Interface, Accessibility, and Interactivity. The one-year experience criterion was adopted as a contextual inclusion criterion rather than a universal measure of respondent reliability. In the present study, we considered at least one year of experience sufficient to provide repeated exposure to online learning activities and platform functions. Prior experience with online learning can also influence students’ perceptions and evaluations of online learning environments. Respondents also reported experience with commonly used platforms, including Moodle, Coursera, and Google Classroom, which provided a practical basis for their pairwise judgments. The respondents also reported experience with commonly used platforms, including Moodle, Coursera, and Google Classroom, which provided a practical basis for their pairwise judgments.

### 3.3 AHP process

The Analytic Hierarchy Process (AHP) is a structured decision-making technique that breaks down complex decisions into smaller, manageable parts. It aids in quantifying subjective criteria through a systematic evaluation process, ultimately ranking decision alternatives based on their relative importance. The key steps involved in the AHP are described below:

#### 3.3.1 Defining the problem

The first step in the AHP process is to clearly define the decision problem. This step is crucial because it lays the groundwork for decision-making. In educational contexts, the problem might involve selecting the best online learning platform based on criteria that influence student engagement and academic performance. The problem should be well formulated to align with the organization’s objectives and decision-making goals.

#### 3.3.2 Structuring the hierarchy

The AHP model was structured into two levels. The first level represented the overall goal: evaluating online learning platforms. The second level consisted of four evaluation criteria: Content Quality, User Interface, Accessibility, and Interactivity. The present study focused on determining the relative importance of these four criteria based on graduate students’ pairwise judgments. Moodle, Coursera, and Google Classroom were considered as representative examples of online learning platforms discussed in the study, but they were not included as alternatives in the AHP calculation. Therefore, the study did not calculate alternative-level priority vectors.

#### 3.3.3 Pairwise comparisons

After establishing the hierarchy, the study compared the four evaluation criteria pairwise. Decision-makers compare the relative importance of each criterion with others, using a scale from 1 (equally important) to 9 (extremely more important). Decision-makers record the comparisons in a matrix, where each element represents the relative importance of one criterion over another. These judgments reflect decision-makers’ expertise or evidence and help determine how each factor contributes to the overall goal. The aggregated pairwise comparison matrix represents the consolidated judgments of all participants. From this matrix, we computed each criterion’s relative weight by normalizing the columns and averaging each row to obtain initial priorities. We then normalized these weights so their total equaled 1.0, as required by the AHP method. Tables 4 and 5 show the detailed values, linking participant responses to the computed priority weights.

#### 3.3.4 Calculating priority vectors

Once the comparisons are made, AHP uses them to calculate priority vectors—numerical scores representing each criterion’s relative importance or each alternative’s relative importance. This is done through matrix calculations, often by finding the normalized eigenvector of the comparison matrix. In our example, the criteria priority vectors assign weights to each factor, indicating its relative importance in the decision-making process. You can also calculate priority vectors for alternatives under each criterion to rank them accordingly.

#### 3.3.5 Consistency ratio (CR) analysis

A critical step in AHP is the Consistency Ratio (CR), which ensures the decision-makers’ judgments are consistent. The CR compares the consistency of pairwise comparisons with that of randomly generated comparisons. If the CR is less than 0.1 (10%), the judgments are considered consistent; if it exceeds this threshold, revisions in pairwise comparisons are necessary to ensure reliability. Steps for Calculating Consistency Ratio:

1. *Compute the consistency index (CI)*:


CI=λmax−1n−1

where $\lambda_{\max}$ is the largest eigenvalue of the matrix, and n is the number of criteria.

2. *Random consistency index (RI)*: Predefined values exist for different-sized matrices (e.g., for 4 criteria, RI = 0.90).

3. *Consistency ratio (CR)*:


$$\mathrm{CR}=\frac{\mathrm{CI}}{\mathrm{RI}}$$

In this study, the largest eigenvalue ( $\lambda_{\max}$ ) was computed directly from the pairwise comparison matrix to determine the Consistency Index (CI) and the Consistency Ratio (CR). Following Saaty’s standard approach, we used the Random Index (RI = 0.90 for *n* = 4). This step ensures that the judgments used to derive weights are reliable and logically coherent.

## 4 Results and discussion

### 4.1 Pairwise comparison and weights of criteria

The values in the pairwise comparison matrix (Table 3) originate from the aggregated judgments of 50 graduate students, whose responses were combined using the geometric mean to form a single representative matrix for analysis. To begin the pairwise comparison, we need to establish the relative importance of each criterion against the others. The pairwise comparison matrix captures these comparisons. Here’s a step-by-step illustration of this process:

#### 4.1.1 Pairwise comparison matrix

We evaluate four criteria: User Interface (UI), Interactivity, Content Quality, and Accessibility. We use the AHP scale, ranging from 1 to 9, to express the relative importance of one criterion over another (Table 3).

In this matrix:

- The diagonal values are all 1, as each criterion is equally important as itself.
- The value of User Interface versus Interactivity is 3, indicating that User Interface is three times more important than Interactivity.
- The value of Interactivity vs. Content Quality is 0.5, indicating Interactivity is less critical than Content Quality.

#### 4.1.2 Calculating priority vectors

To calculate the priority vectors (weights), we followed a systematic procedure to ensure methodological rigor and accuracy. We first normalized the pairwise comparison matrix to convert the raw comparison values to a dimensionless scale, enabling meaningful comparisons across all criteria. Each element of the matrix was divided by the sum of its column, yielding a normalized matrix in which each column sums to 1. This normalization process ensures that each criterion’s influence is proportionally represented relative to the others. The resulting normalized values, presented in Table 4, form the basis for calculating the average of each row, which gives the relative priority (or preliminary weight) of each criterion before consistency verification.

After normalizing the pairwise comparison matrix, the next step in the Analytic Hierarchy Process (AHP) is to calculate the row averages to determine the initial priority vector for each criterion. This process involves summing the normalized values across each row and dividing by the number of criteria (in this case, four). These averages represent the relative priority, or raw weights, of each criterion before normalization. These average values indicate the unscaled importance of the requirements as judged by participants. To ensure methodological accuracy and comparability, the raw weights are then normalized so that their total equals 1.0 (100%), in accordance with AHP requirements (Saaty, 1980). This normalization step ensures that the derived weights accurately reflect the proportional importance of all factors without distortion.

Figure 4 illustrates the relative importance of the criteria evaluated in the study for assessing online learning platforms. The AHP analysis produced four normalized priority weights based on the aggregated pairwise comparison matrix. Content Quality received the highest weight of 0.416 (41.6%), followed by User Interface (0.297, 29.7%), Interactivity (0.147, 14.7%), and Accessibility (0.140, 14.0%). These values indicate the relative importance respondents assigned to the four evaluation criteria. The weights sum to 1.000, confirming the normalization of the priority vector.

**Figure 4:**
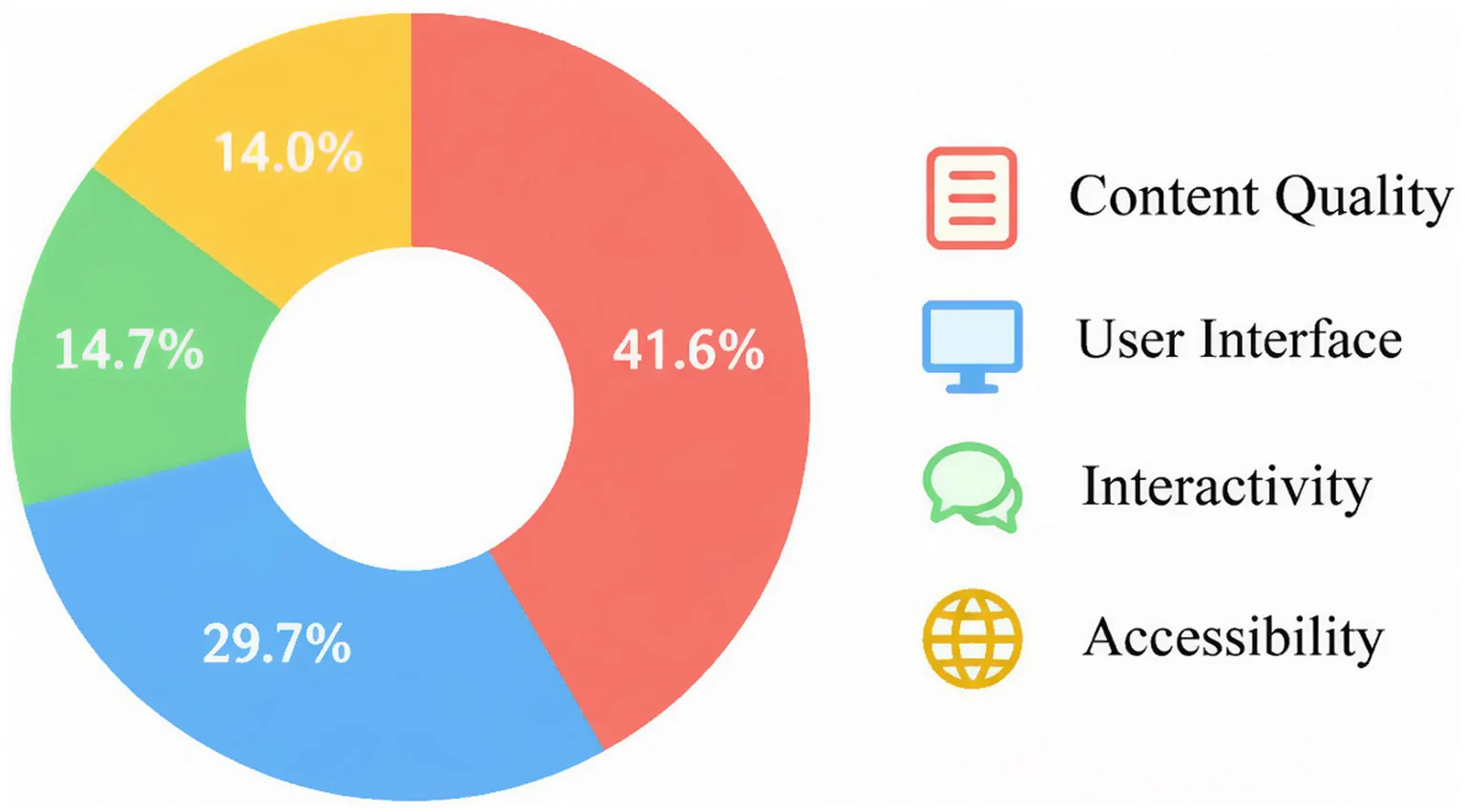
Relative importance of evaluation criteria for online learning platforms based on normalized AHP weights, illustrating the dominance of Content Quality and User Interface in comparison to Accessibility and Interactivity.

### 4.2 Consistency ratio (CR) calculation

To evaluate the reliability of the pairwise comparison judgments, the Consistency Ratio (CR) was recalculated using Saaty’s (1980) eigenvalue method. From the 4 × 4 pairwise comparison matrix (criteria: User Interface, Interactivity, Content Quality, Accessibility), the principal eigenvalue was obtained as $\lambda_{\max}$ = 4.1031. The Consistency Index (CI) was computed using the formula:


$$\mathrm{CI}=\frac{\lambda_{\max}-1}{n-1}$$

where *n* is the number of criteria (*n* = 4), substituting the values gives:


$$\mathrm{CI}=\frac{4.1031-4}{3}=0.0344$$

Using Saaty’s Random Index (RI = 0.90) for *n* = 4, the Consistency Ratio (CR) is:


$$\mathrm{CR}=\frac{\mathrm{CI}}{\mathrm{RI}}=\frac{0.0344}{0.90}=0.038$$

The obtained CR = 0.038 (≈ 0.04) is well below the 0.10 threshold, indicating that the pairwise comparisons were acceptably consistent. Therefore, while not “perfect,” the results demonstrate high reliability and internal coherence in the judgments. A geometric-mean verification method yielded nearly identical weights, confirming the consistency test’s robustness. The AHP analysis identified Content Quality as the most important criterion, with a normalized weight of 0.416, followed by User Interface (0.297), Interactivity (0.147), and Accessibility (0.140). These results indicate the relative importance respondents assigned to the four criteria. These results show that the structure, relevance, and clarity of educational content have the greatest impact on student engagement and performance. High-quality, well-organized content encourages deeper understanding, critical thinking, and sustained motivation. The user interface plays an equally vital role by ensuring intuitive navigation, a clear layout, and a minimal cognitive load, allowing learners to focus on their studies rather than system operation. Accessibility remains essential for providing equitable opportunities, ensuring that students of all backgrounds and abilities can fully engage with learning materials. Although interactivity contributes positively to participation and real-time feedback, it ranks comparatively lower than the foundational factors of content quality, usability, and accessibility. The recalculated Consistency Ratio (CR = 0.038), well below the 0.10 threshold, confirms that the pairwise comparisons were acceptably consistent and methodologically reliable. Overall, these findings suggest that efforts to improve online learning platforms should first focus on strengthening content quality and interface design, then enhance accessibility and interactivity to create more inclusive and engaging learning environments.

### 4.3 Discussion

In our analysis, Content Quality emerged as the most critical criterion, with a weight of 0.54. This finding underscores the significant role that high-quality content plays in online learning environments. According to Moore and Kearsley (2012), educational content quality is a fundamental component of student success and satisfaction. Their research highlights that content aligned with learning objectives significantly enhances the learning experience, consistent with our results (Moore and Kearsley, 2012). Hattie (2008) further supports this view by emphasizing that well-structured, high-quality content promotes critical thinking and better learning outcomes (Hattie, 2008). This aligns with our ranking and reinforces the importance of content quality for effective learning. The User Interface (UI) was ranked second, with a weight of 0.475, reflecting its importance in ensuring ease of navigation and usability. A well-designed UI minimizes cognitive load and lets students focus on the learning material rather than struggling with the platform. Surjono et al. (Surjono et al., 2019) found that an intuitive, user-friendly interface improves student satisfaction and learning efficiency. Their study shows that clear, structured UI designs contribute to better learning experiences, supporting our findings. Additionally, Zhang et al. (Zhang et al., 2004) argue that the user interface design directly affects student engagement and performance. Their research shows that effective UI design facilitates smoother navigation and resource access, aligning with our results. Accessibility, with a weight of 0.3125, ranked third, highlighting its importance in ensuring that all students, regardless of location, device, or disability, can access learning materials effectively. Seale (Zhang et al., 2004) emphasizes that accessibility features are crucial for inclusive education, ensuring that students with disabilities can fully participate in online learning environments. This is consistent with our results, which prioritize accessibility as a key factor in providing equitable learning opportunities. Barteit et al. (Barteit et al., 2020) also support the importance of accessibility in e-learning platforms. Their research suggests that accessible design practices are essential for supporting diverse learner needs and providing equitable opportunities, aligning with our ranking. Interactivity, though ranked lowest with a weight of 0.2, remains essential for enhancing student engagement through features such as quizzes and discussion forums. Croxton (Croxton, 2014) highlighted that interactive elements can significantly improve student engagement and learning. However, their research suggests that while interactivity is beneficial, it may be less critical than content quality, user interface, or accessibility. This aligns with our results, which indicate that while interactivity adds value, it is secondary to content quality, User Interface, and accessibility.

Educational institutions should prioritize integrating high-quality content, user-friendly interfaces, and accessibility features when selecting and designing online learning platforms. To address content quality, institutions should seek platforms that offer well-structured, up-to-date, relevant materials aligned with educational objectives and that support deep learning. A robust content management system that allows for regular updates and easy integration of multimedia resources can enhance the learning experience and keep students engaged. For user interface design, institutions should choose platforms with intuitive navigation and clear layouts to minimize cognitive load and facilitate a seamless learning experience. Features like customizable dashboards and easy-to-access resources can improve usability and student satisfaction. Accessibility features are equally crucial for creating an inclusive learning environment. Platforms should support various assistive technologies and adhere to accessibility standards, ensuring all students, including those with disabilities, can fully engage with the learning materials. Institutions should also consider integrating interactive elements, such as quizzes and discussion forums, but with the understanding that while these enhance engagement, they should complement rather than overshadow the core focus on content quality and usability. By aligning platform selection and instructional design with these priorities, educational institutions can create a more effective, engaging, and equitable online learning experience for all students.

The prominence of Content Quality reflects its central role in ensuring academic rigor and relevance in online learning. This finding aligns with previous research, which found that content clarity and instructional design outweighed interface aesthetics or interactivity in influencing learner satisfaction. However, the comparatively lower weight of Interactivity in this study contrasts with findings from Western studies, which often emphasize interaction as a key driver of engagement. This divergence may stem from contextual differences in learning culture, platform familiarity, and course design approaches. Similarly, Accessibility was valued moderately, suggesting that while inclusivity and ease of access are recognized as important, students prioritize content depth and interface usability once basic accessibility needs are met. Overall, the results reinforce the view that pedagogical quality and usability remain the most critical determinants of online learning effectiveness, consistent with, yet also extending, the insights of earlier MCDM-based evaluations.

From a practical perspective, these findings offer clear guidance for institutions and online platform developers seeking to enhance learning effectiveness. The high weighting of Content Quality suggests that universities should prioritize developing and curating course materials that are pedagogically sound, regularly updated, and aligned with learning outcomes. Investment in professional instructional design and multimedia integration can further increase content engagement and comprehension. The strong influence of User Interface (UI) underscores the importance of intuitive navigation, aesthetic clarity, and responsive design, which collectively reduce cognitive load and improve user satisfaction. Emphasizing Accessibility requires ensuring compatibility with assistive technologies, flexible delivery formats, and low-bandwidth options to accommodate diverse learners. Although Interactivity ranked lowest, it remains an important secondary factor that can enhance learner motivation through features such as discussion forums, quizzes, and collaborative tools. By addressing these dimensions in proportion to their relative importance, institutions can create more effective, inclusive, and user-centered online learning ecosystems that directly respond to student needs.

### 4.4 Limitations of the study

*Institutional Generalizability*: The sample consists of 50 graduate students from Guangxi Minzu Normal University in China and was obtained through convenience sampling. Although respondents represent four academic disciplines, the sample is limited to a single institution. Therefore, the findings may not be generalizable to students from other institutions, educational systems, or cultural contexts.

*Subjective Assessment of Academic Performance*: Although academic performance is included in the title, abstract, and research objectives, the study does not include objective academic performance indicators such as examination grades, grade point average, or course completion rates. The analysis is based on students’ perceptions of the factors that may influence their academic performance. Therefore, the findings represent perceived importance rather than a direct relationship between the evaluation criteria and measured academic outcomes.

*Limited Evaluation of Platform Alternatives*: Moodle, Coursera, and Google Classroom were selected as representative examples of online learning platforms discussed in this study. These platforms were not included as alternatives in the AHP calculation. The AHP analysis was limited to determining the relative importance of the four evaluation criteria. Consequently, the study does not provide an empirical AHP ranking of Moodle, Coursera, and Google Classroom. Future research should extend the AHP hierarchy by evaluating each platform under each criterion and calculating the corresponding alternative priorities.

## 5 Conclusion

The acceptable consistency ratio confirms the reliability of the derived priorities. These findings suggest that the structure, clarity, and pedagogical value of the content, combined with a straightforward, user-friendly interface, are the most significant factors influencing the effectiveness of online learning platforms. Accessibility features that enable equitable participation remain important, while interactivity contributes positively but with comparatively lower weight in the overall evaluation. The corrected results strengthen the analysis’s validity and provide a transparent foundation for further empirical verification. Future investigations should replicate the procedure across larger and more diverse samples to confirm the stability of the weighting structure and broaden the applicability of these insights to other educational contexts.

Beyond replicating earlier AHP applications, this research provides a novel contextual and methodological contribution. It extends AHP to a diverse graduate student population in a non-Western higher education setting, providing empirical evidence on how cultural and institutional factors influence preferences for online learning platform design. The study also demonstrates a transparent computation process and a verified consistency ratio, reinforcing methodological integrity and providing a framework that future studies can adapt and expand. These findings offer practical guidance for universities and platform developers, suggesting that investments should prioritize instructional content design and user interface quality before expanding interactive features.

Future research should extend the present study by using larger and more diverse samples from multiple universities and educational contexts. Probability-based sampling can be considered to improve the representativeness of the respondents. Future studies should also include domain experts and compare student and expert judgments to examine differences in the perceived importance of the four criteria. In addition, fuzzy AHP or an integrated AHP TOPSIS approach can be applied to examine uncertainty in pairwise judgments and evaluate specific online learning platforms. Cross-institutional and cross-country studies can further examine whether the relative importance of Content Quality, User Interface, Accessibility, and Interactivity changes across educational and cultural contexts. Longitudinal studies can also examine whether these priorities change with increased experience with online learning platforms.

## Data Availability

The data analyzed in this study is subject to the following licenses/restrictions: The dataset supporting the findings of this study is available from the corresponding author upon reasonable academic request. Requests to access these datasets should be directed to Guanlei Yin; gll578508@gmail.com.
